# The Influence of Continuous Exercising on Chronotropic Incompetence in Multi-Episode Schizophrenia

**DOI:** 10.3389/fpsyt.2019.00090

**Published:** 2019-03-13

**Authors:** Marco Herbsleb, Katriona Keller-Varady, Thomas Wobrock, Alkomiet Hasan, Andrea Schmitt, Peter Falkai, Holger Horst Werner Gabriel, Karl-Jürgen Bär, Berend Malchow

**Affiliations:** ^1^Psychiatric Brain and Body Research Group, Department of Psychiatry and Psychotherapy, University Hospital Jena, Jena, Germany; ^2^Department of Sports Medicine and Health Promotion, Friedrich-Schiller-University of Jena, Jena, Germany; ^3^Hannover Medical School, Institute of Sports Medicine, Hannover, Germany; ^4^Department of Psychiatry and Psychotherapy, County Hospitals Darmstadt-Dieburg, Groß-Umstadt, Germany; ^5^Department of Psychiatry and Psychotherapy, University Hospital LMU, Munich, Germany; ^6^Laboratory of Neuroscience (LIM27), Institute of Psychiatry, University of São Paulo, São Paulo, Brazil

**Keywords:** schizophrenia, exercise, chronotropic incompetence, cardiac autonomic dysfunction, heart rate response

## Abstract

People with schizophrenia die on average 15–20 years earlier than age and gender matched controls in the general population. An essential part of this excess mortality in people with schizophrenia is caused by physical illnesses. Among the physical illnesses, cardiovascular disease (CVD) has been identified as the most common natural cause of death in up to 40–45% of the cases. Chronotropic incompetence (CI) is defined as the inability of the heart to increase its beating frequency in proportion to increased physical activity or higher metabolic demand. It is an established independent cardiovascular risk factor for major cardiac events and overall mortality and might explain adaptation intolerance of the cardiovascular system to even minor exercise courses. CI needs objective exercise testing for definitive diagnosis and therefore represents a biological marker indicating the integrity of the cardiovascular system. It was recently described in patients with schizophrenia and might help explain the reduced physical fitness in these patients and the inability of a subgroup of patients to benefit from exercise interventions. In this study, we tried to replicate the occurrence of CI in an independent sample of patients with schizophrenia and evaluated whether CI can be influenced by a continuous endurance training of 12 weeks. Therefore, we re-analyzed the fitness testing data of 43 patients with schizophrenia and 22 aged and gender matched healthy controls. Parameters of aerobic fitness and chronotropic response to exercise were calculated. Patients with schizophrenia were less physically fit than the healthy controls and displayed a significantly higher heart rate at rest. 10 of 43 patients with schizophrenia and no healthy control subject were classified as chronotropically incompetent. Chronotropic response to exercise did not change significantly after 12 weeks of continuous aerobic exercise training. No differences were observed for baseline heart rate and peak heart rate in both subgroups of schizophrenia patients. Aerobic fitness did not improve significantly in the patients with schizophrenia classified as chronotropically incompetent. Our results confirm the occurrence of CI in patients with multi-episode schizophrenia. This should be taken into account when planning an exercise or lifestyle intervention studies in this population. Schizophrenia patients with CI do not seem to benefit as well as schizophrenia patients without CI from aerobic exercise training interventions. Larger, prospective randomized controlled clinical trials with different training interventions are urgently needed to address the topic of schizophrenia patients not responding to exercise and the relationship to the illness itself.

## Introduction

There is a solid body of evidence underpinning the notion that people with schizophrenia die on average 15–20 years earlier than age and gender matched controls in the general population (1–3). This mortality gap between the general population and patients with schizophrenia seems to increase instead of converge over the last decades (4–6). An essential part of this excess mortality in people with schizophrenia is caused by physical illnesses (7–9). In several studies, cardiovascular disease (CVD) has been identified as the most common natural cause of death in up to 40–45% of cases (7, 10). It has been suggested that coronary heart disease is the main contributor to CVD, which is exacerbated by the high-risk profile (obesity, poor diet, lack of exercise, smoking) of most patients with schizophrenia. In addition, patients with schizophrenia take antipsychotic medication on a regular basis, which is known to affect the cardiovascular system additionally (11). Some of the risk factors are potentially reversible by changing the lifestyle and even by considering side effects of some antipsychotics. A non-pharmacological interventional approach to impact cardiovascular risk factors is regular physical exercise (12, 13). As mentioned above, studies have shown that people with severe mental illness like schizophrenia engage in significantly more sedentary behavior and significantly less physical activity compared to healthy controls (14). Additionally to somatic health benefits, aerobic exercise positively impacts core features of the disease itself like cognitive deficits (15–17) and negative symptoms (18). However, although the above-mentioned risk factors seem to be plausible they do not explain the excess mortality of patients with schizophrenia satisfactorily. Moreover, psychiatrists and researchers seem to overlook to some extend the solid body of evidence describing cardiovascular dysregulation in patients with schizophrenia both at rest and during physical activity. It has been shown that young unmedicated patients with schizophrenia show increased heart rates at rest caused by severe autonomic dysfunction (19). An increase in heart rate of 5 beats per minute corresponds to a significant increase in the atherosclerosis progression (20). Life expectancy in animals and humans shows a close relation to the medium heart rate (21). Increased resting heart rate has been shown to be a risk factor for reduced life expectancy in both the general population (22, 23) and in populations with cardiovascular diseases (24, 25). In patients with schizophrenia, the protective vagal component seems to be profoundly impaired leading to reduced heart rate variability or an insensitive baroreflex. While similar findings have been described in patients with depression (26), alcohol withdrawal (27), or anxiety disorders (28) the pronounced degree of changes leads to a unique pattern in patients with schizophrenia. Most of the described alterations in the autonomic nervous system are associated with reduced life expectancy previously shown in various conditions such as diabetes (29), survival after myocardial infarction (30, 31) or in cardiac artery disease (CAD) (32). Thus, more research is needed to better explain cardiovascular dysregulation in schizophrenia because in contrast to patients suffering from severe CAD the cardiac autonomic dysfunction in schizophrenia is not caused by structural or functional alterations of the heart itself but moreover by abnormalities in the brain—heart interaction (33).

More recently, we investigated the putative link between autonomic dysfunction and physical incapacity in these patients. And indeed, Herbsleb et al. (34) were able to present evidence that about half of the tested population of patients with schizophrenia showed a strikingly absent heart rate acceleration during physical exercise. This phenomenon is called chronotropic incompetence of the heart (CI) and was again described in various cardiac conditions (35–38). It is defined as the inability of the heart to increase its beating frequency in proportion to the increase in physical activity or higher metabolic demand and therefore needs an exercise testing of the patients for definitive diagnosis (37). This finding is of paramount importance since CI is a well-described cardiac risk factor of mortality (39–43), it is associated with physical incapacity and might explain to some extend that patients with schizophrenia encounter difficulties to enjoy physical activities. However, this new finding warrants further studies to identify patients at risk and to investigate treatment options.

Therefore, the aim of the current investigation was to examine if the previously revealed impaired heart rate response to physical exercise is a consistent finding, which can be also detected in a further cohort of patients with schizophrenia. Here, we used a different exercise test protocol in order to elucidate the possibility to include data from various studies and to generalize findings. In addition, we evaluated, whether heart rate responses to exercise can be influenced by a moderate-intensity continuous endurance training of 12 weeks in our patients.

We hypothesized that (i) our re-analysis of fitness testing data would reveal CI in a substantial portion of patients with multi-episode schizophrenia, (ii) that CI is again related to reduced physical fitness in this subset of patients, and (iii) that an continuous aerobic exercise training ameliorates or reverses the CI of the heart in the subset of schizophrenia patients affected.

## Materials and Methods

### Subjects

The evaluated cohort consisted of forty-three multi-episode schizophrenia patients and twenty-two healthy controls who successfully completed an interventional, single center continuous aerobic exercise study which took place at the Department of Psychiatry and Psychotherapy at the University Medical Center Göttingen between 2010 and 2013 (44–48). The local ethics committee approved the study protocol, which was in accordance with the Declaration of Helsinki. All participants provided written informed consent prior to inclusion in the study. The trial was registered at www.clinicaltrials.gov (NCT01776112). Briefly, 43 patients with multi-episode schizophrenia and 22 healthy controls were assessed for endurance capacity using an incremental exercise test on a bicycle ergometer before and after the study intervention. The study intervention consisted of standardized continuous endurance training on cycle ergometers three times a week for 30 min over a total intervention period of 12 weeks. A group of 21 patients with schizophrenia played table soccer (also known as foosball is the US) as an additional active control intervention. Healthy controls were matched regarding age and gender to patients in the endurance group (see Table 1). Inclusion criteria were a diagnosis of schizophrenia according to ICD-10, stable psychopathology and antipsychotic medication for 2 weeks prior to the intervention and age between 18 and 60 years. Exclusion criteria were substance abuse (alcohol and illicit drugs (cannabis, amphetamines, cocaine, benzodiazepines, and opioids) assessed with urine drug tests), a worsening of psychopathological symptoms within 2 weeks of the screening period, pregnancy, lactation, or contraindications for endurance training or maximal cardiopulmonary exercise testing (CPET).

**Table 1:** Baseline characteristics as well as parameters of aerobic fitness in the control and patient group.

**Baseline characteristic**	**Controls**	**Patients**	***P*-value**
	***n* = 22**	***n* = 43**	
**EPIDEMIOLOGICAL DATA**			
Age; years	37.6 ± 11.3	36.3 ± 12.9	0.589
Gender: male/female	16/7	31/12	0.743
Body mass; kg	80.8 ± 13.6	90.4 ± 19.2	0.052
BMI; kg/m^2^	26.0 ± 3.7	28.4 ± 4.9	**0.023**
**DISEASE SPECIFIC DATA**			
Duration of disease (years)	NA	10.9 ± 9.3	-
PANSS	NA	69.3 ± 6.3	-
**MEDICATION**			
Chlorpromazine equivalents, CPE *(mg/d)*	NA	658 ± 674	
Clozapin (yes/no)	NA	5/38	
**PARAMETERS OF AEROBIC FITNESS**			
Peak oxygen uptake; ml/kg/min	35.9 ± 7.5	29.5 ± 8.7	**0.011**
Peak oxygen uptake; % pred	93 ± 22	113 ± 26	**0.001**
Peak power output; W/kg	2.54 ± 0.48	1.87 ± 0.54	**<0.001**
PWC 130; W/kg (patients: *n* = 40)	1.32 ± 0.36	1.09 ± 0.32	**0.013**
PWC 150; W/kg (patients: *n* = 42)	1.76 ± 0.38	1.44 ± 0.44	**0.009**
Power output at Lactate Threshold; W/kg	1.28 ± 0.24	1,00 ± 0.32	**0.001**
**OBJECTIVE EFFORT**			
Peak blood lactate, mmol/l	9.6 ± 2.7	7.6 ± 2.6	**0.005**
**HEART RATE DATA AND CHRONOTROPIC RESPONSE**			
Baseline heart rate; 1/min	78 ± 10	90 ± 15	**0.001**
Peak heart rate; 1/min	180 ± 14	171 ± 18	0.069
% pred. heart rate reserve	98 ± 12	88 ± 18	**0.017**
Slope metabolic-chronotropic relationship	1.05 ± 0.11	0.91 ± 0.20	**<0.001**
Chronotropic incompetence (yes/no)	0/22	10/33	**0.014**

*Data are shown as mean ± standard deviation or absolute number*.

*n, number of participants if not all are include in calculation because of excluded or missing data (e.g., failure to reach a given heart rate); NA, not applicable*.

*Bold values indicate significant differences between groups*.

### Exercise Testing

In order to measure aerobic endurance capacity before and after the 12-week intervention, participants performed incremental CPET until subjective exhaustion on an electronically braked bicycle ergometer (Ergoselect 200 K, Ergoline GmbH, Bitz, Germany). After a resting period of 3 min, the incremental bicycle protocol started at a power output of 25, 50, or 75 W on the basis of the anamnestic information for 3 min. The power output was then increased in steps of 25 W every 3 min until the subject reached his or her limit of tolerance. During the CPET, ventilatory indices and gas exchanges and heart rate were measured continuously on a breath-by-breath basis using an automatic mobile wireless ergospirometer (MetaMax 3B, Cortex, Biophysik GmbH, Leipzig, Germany, and T31, Polar Electro Oy, Kempele, Finland). Before each test, the turbine (flow and volume) was calibrated with a syringe (Hans Rudolph Inc., Kansas City). For data analysis, the breath-by-breath gas exchange and ventilatory data were smoothed using a 15-breath moving average, aligned to the time of the central breath. Peak oxygen consumption (VO_2_peak) was defined as the highest value of 15-breath average occurring during CPET. Capillary whole-blood samples of 0.2 μl were obtained for lactate measurements from the ear lobe when the patient was at rest, and again at the end of the third minute of each 3-min period of the CPET. The blood lactate concentrations were measured by Lactate SCOUT Solo Plus, SensLab GmbH, Leipzig, Germany).

### Parameters of Aerobic Fitness

A set of different physiological maximal and submaximal indices was used to describe the aerobic fitness. We calculated the power output at a fixed heart rate of 130 and 150 (known as physical working capacity; PWC 130, PWC 150), as well as the power output at the Lactate Threshold (LT), which are both submaximal indices of aerobic fitness. The time course of lactate was graphically interpolated using an equalizing spline procedure. The LT was defined as the minimum lactate equivalent (the lowest value of the quotient lactate to power output) as describes the first sustained increase in blood lactate concentration above resting level during incremental exercise (49). The evaluation of the LT and the power output achieved at a blood lactate concentrations of 2 mmol/l (for training prescription) were done examiner independent by using a software for performance diagnostic (Ergonizer, Germany, www.ergonizer.de). Moreover, we determined the achieved peak power output (Ppeak) and peak oxygen uptake (VO_2_peak). More details for the calculation of the PWC, VO_2_peak, and Ppeak are given in Keller-Varady et al. (45).

### Continuous Endurance Training

The interventions in the patients with schizophrenia consisted of a 12-week training procedure. Participants participated in three training sessions per week lasting exactly 30 min each. The sessions consisted of moderate-intensity endurance training on cycle ergometers. The exercise intensity was set to the individual power output achieved at a blood lactate concentrations of 2 mmol/l, determined during the preceding CPET. Power output was than increased gradually, corresponding to improvements in endurance performance, by a mean of 8 ± 1 % after 11 ± 6 training sessions, controlled by lactate concentration monitoring.

### Assessment of Heart Rate Responses to Exercise

To assess chronotropic response, the metabolic-chronotropic relationship (MCR; also known as the chronotropic index) was calculated according to (50) using the ratio of heart rate reserve (predicted HRpeak (220—age)—HR at rest) to metabolic reserve (VO_2_peak—VO_2_ at rest) during the CPET. The MCR adjusts for age, physical fitness and functional capacity and is unaffected by the exercise protocol as well as effort-independent (37). The increase in percentage heart rate reserve in relation to the increase in percentage metabolic reserve (also known as oxygen uptake reserve) was obtained by linear regression analysis using the least squares method of the mean values acquired for VO_2_ and HR data in the last 30 s of each incremental step of the CPET. In healthy adults, the percentage of heart rate reserve achieved during exercise equals the percentage of metabolic reserve achieved, so that the median MCR slope is near around 1.0 with a 95% confidence interval between 0.8 and 1.3 (50).

### Statistics

For statistical analysis, SPSS for Mac (version 25.0.0.1) was used. All parameters were tested for normal distribution with the Kolmogorov–Smirnov test or with Shapiro-Wilk-Test by subgroup analysis. Epidemiological data and objective effort during exercise testing of patients and controls as well as subgroups of patients (CI vs. non CI patients) were compared by either using parametric or non-parametric two-sample tests as well as Chi-squared tests for comparison of nominal data. To investigate basic differences of aerobic fitness and heart rate parameters between all patients and controls, we performed two separate multivariate analyses of variance (MANOVAs). We conducted a first MANOVA for aerobic fitness parameters with the inter-subject factor GROUP (patients vs. controls) including VO_2_peak, Ppeak, PWC 130, PWC 150, as well as LT (all normalized to body mass) followed-up by univariate analysis of variances (ANOVAs). The second MANOVA for all participants was performed by applying the factor GROUP (patients vs. controls) and included the following heart rate parameters: baseline heart rate, peak heart rate, and percentage of the predicted heart rate reserve (% pred. heart rate reserve). ANOVAs were then calculated for single parameters. To investigate the main hypothesis of our study, the metabolic–chronotropic relationship slope (MCR slope) of patients and controls was analyzed. Controls and patients with a MCR slope below the cut-off value of 0.8 were referred as chronotropic incompetent whereas subjects with a MCR slope above a value of 0.8 were classified as chronotropic competent (50). Group differences of the MCR slope between patients and controls were verified with a parametric two-sample *t*-test. We repeated a MANOVA with respect to physical fitness for the factor SUBGROUP (CI vs. nonCI patients) followed-up by univariate ANOVAs. Due to the influence of chronotropic incompetence on the heart rate derived fitness indices PWC 130 and PWC 150, we abstained form including these parameters in the analysis. The effects of the continuous aerobic endurance training on aerobic fitness indices (including VO_2_peak, Ppeak, PWC 130, PWC 150, and LT) in subgroups of patients (CI vs. nonCI) were examined using the dependent sample *t*-test or the Wilcoxon matched pairs signed rank test as the nonparametric alternative. We applied the same procedure to investigate the effects of training MCR-slope in CI and non-CI patients. The significance level was set 0.05 (two-sided) and fitted using Bonferroni adjustment procedure to account for inflation of alpha level by multiple testing.

## Results

### Physical Fitness

The MANOVA for aerobic fitness indices (VO_2_peak, Ppeak, PWC 130, PWC 150, LT) revealed a significant difference [*F*_(5, 56)_ = 5.66; *P* < 0.001] between all schizophrenia patients and healthy controls. The follow-up univariate ANOVAs indicated significant differences for VO_2_peak (*F* = 6.93; *P* < 0.05), Ppeak (*F* = 21.84; *P* < 0.001), PWC 130 (*F* = 6.57; *P* < 0.05), PWC 150 (*F* = 7.35; *P* < 0.01 and LT (*F* = 11.38; *P* < 0.01; see Table 1).

### Heart Rate

The second MANOVA for heart rate parameters revealed a significant effect for the factor GROUP [*F*_(3, 61)_ = 6.45; *P* < 0.01]. As depicted in Table 1, the ANOVAs showed significant differences between all patients and controls for baseline heart rate (*F* = 11.60, *p* < 0.01) and percentage of predicted heart rate reserve (*F* = 6.02, *P* < 0.05), whereas only a trend was found for peak heart rate (*F* = 3.42, *P* < 0.069).

### Metabolic–Chronotropic Relationship

**Figure 1:**
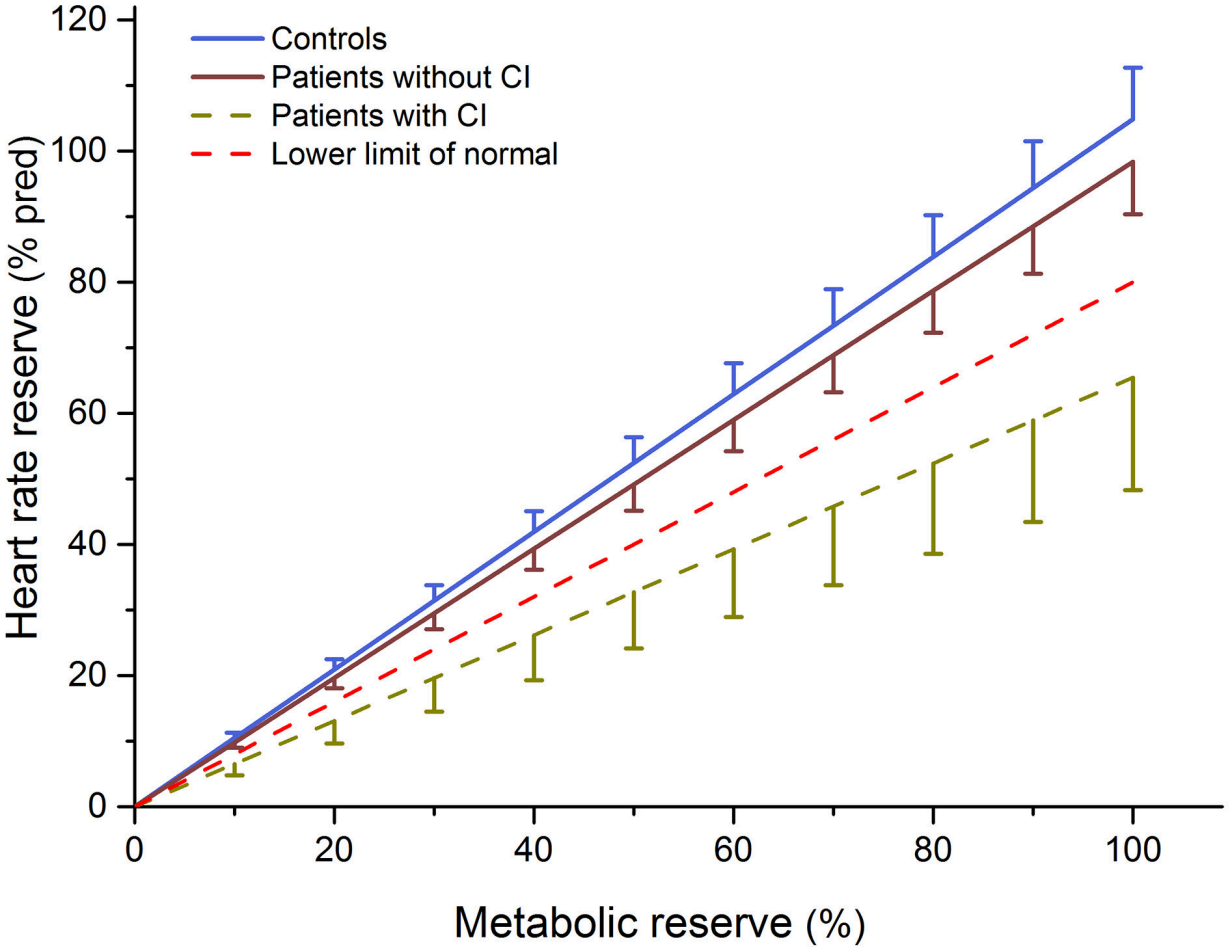
Mean ± 90% confidence limit of the metabolic–chronotropic relationship of patients with (CI) and without (non-CI) chronotropic incompetence as well as control subjects. The dotted line represents the lower limit of normal (slope < 0.8).

**Figure 2:**
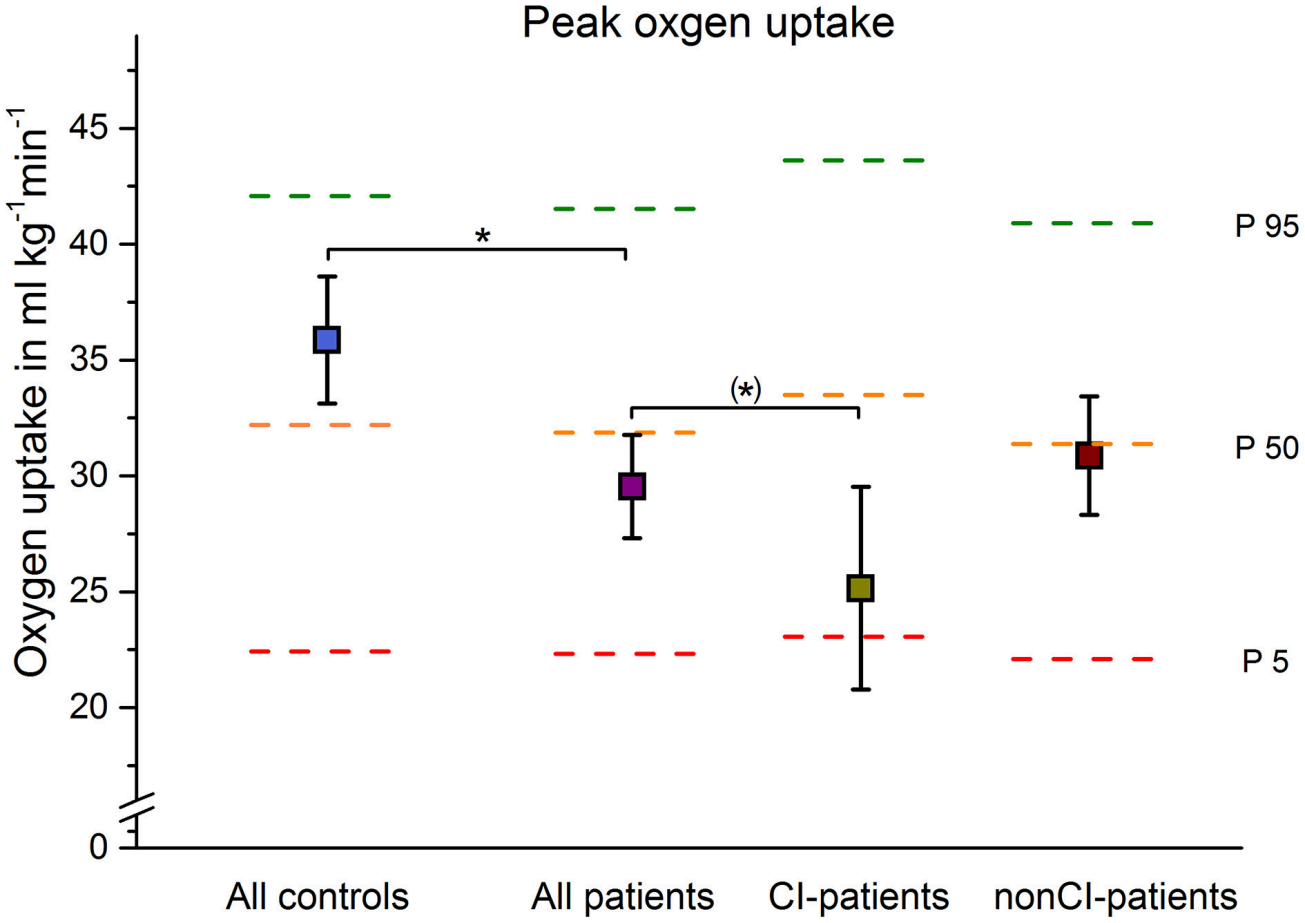
Mean ± 90% confidence limit of peak oxygen uptake of all patients, and patients with (CI) and without (non-CI) chronotropic incompetence are depicted in comparison to controls. The dotted horizontal lines represent contemporary age, gender, and body size specific reference percentiles (51). Significant or a trend differences are indicated by *p < 0.05 or (*) *p* < 0.1, respectively.

Based on cut-off values by Burbaker and Kitzman, (37) and Wilkoff and Miller, (50), 10 of 43 patients with schizophrenia had an impaired heart rate response to exercise and were classified as chronotropically incompetent, whereas no healthy control subject was below the cut-off value. Furthermore, a significant difference was observed between the mean MCR slopes of patients and controls (*P* < 0.001, see Table 1 and Figure 1). The MANOVA that compared aerobic fitness indices (VO_2_peak, Ppeak, and LT) between subgroups of patients (CI vs. nonCI patients) revealed no significant difference between groups [*F*_(3, 39)_ = 1.76; *P* = 0.170] (see Table 2 and Figure 2).

**Table 2:** Baseline characteristics as well as parameters of aerobic fitness in patients with schizophrenia with (SZ-CI) and without chronotropic incompetence (SZ-nonCI).

**Parameters**	**NonCI-patients**	**CI-patients**	***P*-value**
	***n* = 33**	***n* = 10**	
**EPIDEMIOLOGICAL DATA**			
Age; years	37.4 ± 13.5	34.0 ± 11.3	0.542
Gender: male/female	22/11	9/1	0.150
Body mass; kg	87.2 ± 15.4	101 ± 27	0.204
BMI; kg/m^2^	27.6 ± 4.1	31.3 ± 6.2	**0.033**
**DISEASE SPECIFIC DATA**			
Duration of disease (years)	10.7 ± 9.8	11.6 ± 8.0	0.702
PANSS	70.0 ± 6.6	67.1 ± 5.5	0.724
**MEDICATION**			
Chlorpromazine equivalents, CPE (mg/d)	624 ± 632	767 ± 822	0.687
Clozapin (yes/no)	2/31	3/7	**0.039**
**PARAMETERS OF AEROBIC FITNESS**			
Peak oxygen uptake; ml/kg/min	30.9 ± 8.6	25.2 ± 7.5	0.067
Peak oxygen uptake; % pred	98 ± 21	74 ± 19	**0.003**
Peak power output; W/kg	1.97 ± 0.52	1.54 ± 0.49	**0.027**
Power output at Lactate Threshold; W/kg	1.06 ± 0.33	0.83 ± 0.21	**0.017**
PWC 130; W/kg (CI: *n* = 9; nonCI: *n* = 31)	1.08 ± 0.33	1.14 ± 0.29	0.601
PWC 150; W/kg (CI: *n* = 9)	1.39 ± 0.45	1.61 ± 0.34	0.188
**OBJECTIVE EFFORT**			
Peak blood lactate, mmol/l	7.9 ± 2.6	6.4 ± 2.3	0.089
**HEART RATE DATA AND CHRONOTROPIC RESPONSE**			
Baseline heart rate; 1/min	88 ± 15	92 ± 7	0.629
Peak heart rate; 1/min	177 ± 14	154 ± 17	**<0.001**
% pred. heart rate reserve	95 ± 13	64 ± 13	**<0.001**
Slope metabolic-chronotropic relationship	0.98 ± 0.14	0.65 ± 0.15	**<0.001**

*Data shown as mean ± standard deviation or absolute numbers*.

*n, number of participants if not all are include in calculation because of excluded or missing data (e.g., failure to reach a given heart rate)*.

*Bold values indicate significant differences between groups*.

### Influence of the Moderate Intensity Continuous Aerobic Exercise Training on Heart Rate Response to Exercise

The MCR-slope as an index of chronotropic response to exercise did not change significantly in both, the CI and the non-CI subgroup after 12 weeks of continuous aerobic exercise training compared to baseline (see Table 3). Furthermore, no differences were observed for baseline heart rate and peak heart rate in both groups.

**Table 3:** Differences to baseline in disease specific data, indices of aerobic fitness, and heart rate data after a 12-week moderate-intensity aerobic training in patients without (SZ-nonCI) and with (SZ-CI) chronotropic incompetence.

	**Training nonCI-patients**	**Training CI-patients**
	***n* = 16**	***n* = 6**
**EPIDEMIOLOGICAL DATA**		
Body mass; kg	−0.9 ± 5.2	−0.5 ± 9.1
BMI; kg/m^2^	−0.2 ± 1.6	0.0 ± 2.6
**DISEASE SPECIFIC DATA**		
PANSS	−8.9 ± 18.6*	7.0 ± 13.5
**MEDICATION**		
Chlorpromazine equivalents, CPE (mg/d*)*	−2 ± 473	212 ± 552
**PARAMETERS OF AEROBIC FITNESS BEFORE AND AFTER TRAINING**		
Peak oxygen uptake; ml/kg/min	1.52 ± 5.24	0.45 ± 5.42
Peak oxygen uptake; % pred	4.8 ± 15.7	2.0 ± 16.0
Peak power output; W/kg	0.18 ± 0.23*^#^	0.09 ± 0.43
PWC 130; W/kg (non CI: *n* = 15, CI: *n* = 5)	0.20 ± 0.26*^#^	−0.00 ± 0.37
PWC 150; W/kg (CI: *n* = 5)	0.22 ± 0.26*^#^	0.08 ± 0.43
Power output at Lactate Threshold; W/kg	−0.02 ± 0.32	0.05 ± 0.24
**OBJECTIVE EFFORT**		
Peak blood lactate; mmol/l	0.83 ± 1.66	0.83 ± 2.83
**HEART RATE DATA AND CHRONOTROPIC RESPONSE**		
Baseline heart rate; 1/min	−4.7 ± 13.4	−6.1 ± 7.1
Peak heart rate; 1/min	0.0 ± 10.4	−0.5 ± 18.8
% pred. heart rate reserve	0.1 ± 13.1	3.4 ± 17.0
Slope metabolic-chronotropic relationship	0.01 ± 0.14	0.02 ± 0.20

*Data are shown as mean ± standard deviation*.

*n = number of participants if not all are include in calculation because of excluded or missing data (e.g., failure to reach a given heart rate)*,

* and # indicates statistically significant difference from baseline values, P < 0.05 (^*^not adjusted/^*^^#^adjusted for multiple testing).

*Bold values indicate significant differences before and after training within a group*.

### Responses of Aerobic Fitness to Continuous Aerobic Exercise Training

Of the 22 schizophrenia patients who underwent the 12-week continuous aerobic exercise training intervention, 6 were classified as chronotropic incompetent based on the MCR-Slope as described above. The epidemiological data, aerobic fitness and heart rate parameters are highlighted in Table 3. Aerobic fitness improved significantly (as indicated by Ppeak, PWC 130, PWC 150) in the non-CI group, but contrary to our initial hypothesis it did not change significantly in the CI-group (see Table 3). However, no significant responses to training in both groups were observed for VO_2_peak and LT.

## Discussion

Our study shows in agreement with previous reports that healthy controls show better fitness levels than patients with schizophrenia, despite being age and gender matched and engaging in no sports 2 years before entering the study (45). In addition, this study confirmed in 10 out of 43 patients the pattern of chronotropic incompetence in patients with schizophrenia and thus corroborates the first description in the disease although studied in a different fitness test design. Most intriguingly, we did not observe a significant impact of a twelve-week moderate continuous exercise training on CI values in patients.

In the present study, we identified impaired chronotropic responses with a step-wise incremental exercise test in contrast to the ramp-wise protocol used in the previous study by Herbsleb et al. (34). However, in this independent sample only 23% of the multi-episode patients with schizophrenia showed CI. This is lower than in our previous study where the proportion of SZ-CI consisted of 43% (34). The difference to higher share of CI cases in our previous study may relate to the composition of groups rather than other causes given the small sample size. It seems not plausible that the cause of the higher occurrence of CI in the study of Herbsleb et al. (34) is mainly explained by the different exercise test protocols that were used. Although the ramp-wise protocol results in lower mean HR as well as VO_2_ values at the common power output than step-wise tests [(52), the impact of the test protocol on the MCR slope (which is the relationship of HR and VO_2_ values) is considered to be negligible. A variety of exercise testing protocols and modes of testing have been used to evaluates the MCR relationship (37) since the originally description by Wilkoff and Miller (50), that used a step-wise incremental treadmill exercise test. However, in order to adequately assess the chronotropic response to metabolic stress of exercise the following points should be considered in future study designs: (i) participants should be encouraged to exercise until the subject reaches his or her limit of tolerance, (ii) exhaustion levels should be objectively evaluated by either measuring blood lactate values or the respiratory exchange ratio (ratio of carbon dioxide output to oxygen uptake), (iii) a sophisticated gas-exchange analysis is needed or specific test protocols (e.g., Bruce treadmill protocol, Chronotropic Assessment Exercise Protocol) should be applied and iv) a step-wise incremental exercise test with stage durations ≥3 min is recommended to obtain a near steady-state heart rate and to satisfy the equations conditions.

Since CI is known to be a cardiac risk factor larger trials might even elucidate possible clinical key factors associated with CI. To date, we were unable to find a substantial difference between CI and non-CI patients with respect to psychopathology, body mass index, duration of disease or the prescribed medication (see Table 1). However, it should be noted that patients taking clozapine were not included in the first analysis and the putative influence of clozapine on cardiovascular regulation should be investigated separately in future studies.

Various pathophysiological mechanisms have been proposed leading to chronotropic incompetence in cardiac conditions. For instance, Bristow and colleagues found a 50% or greater reduction in β-adrenergic receptor density in the left ventricular myocardium of failing hearts explanted during transplant surgery (53). These and other findings suggest that in heart failure patients, a decrease in β-receptor density leads to a diminished sensitivity of the β-adrenergic pathway and a decrease in β-agonist–stimulated muscle contractility. We would like to speculate that the long lasting augmented sympathetic drive might lead to a reduction of β-receptor density in patients with schizophrenia. In addition, we cannot exclude that the chronic stress of a multi-episode disease such as schizophrenia might be associated with reduced excretion of catecholamines during an exercise bout. Although we have not observed a difference between both patient groups with respect to medication (34), we cannot completely exclude that anti-dopaminergic activity of some antipsychotics might influence the acute stress response. Since the definite pathophysiology of schizophrenia remains unclear, concepts of neuroinflammation and specifically autoimmune encephalitis or autoimmune psychosis have again gained more ground recently (54–57). However, the definite contribution is still discussed controversially, especially in those cases with no neurological symptoms (58). For the diagnosis of Autoimmune-Encephalitis clinical, imaging and CSF criteria have been proposed (59). Autoimmune antibodies are known to cause autonomic dysfunction in a lot of diseases like Sjögren syndrome or Guillain–Barré syndrome but also in conditions called pure autonomic failure (60). To the best of our knowledge the patients with multi-episode schizophrenia included in this study showed no yellow or red signs pointing to autoimmune etiology (61). Nevertheless, autoimmune antibodies were not studied specifically in the cerebrospinal fluid prior to inclusion in the study and hence cannot be ruled out to cause or promote autonomic dysfunction, here described as CI, in a subgroup of the patients. Nevertheless, CI could be a relatively easy to obtain objective biological marker to study the conjunction with inflammation pathways. Thus, this should be elucidated in further studies in this field and shift the focus on pathophysiological mechanisms to understand regulatory problems in patients with schizophrenia. This might help to decrease some obstacles impeding patients to engage in physical activities on a regular basis and to benefit from it (62). If CI is of importance in a substantial subset of patients with schizophrenia then one needs to think about other routines to accustom patients with schizophrenia to regular physical activity.

It is therefore intriguing that 12 weeks of continuous aerobic exercise training had no impact on CI measures in this study and that patients with multi-episode schizophrenia and CI did not benefit from the regular aerobic exercise in terms of fitness parameters or heart rate at rest. More studies are urgently needed to learn how to modulate the cardiac risk factor CI in patients with schizophrenia. One needs to consider different exercise protocols, longer durations of exercise and adjustments as well as an optimal assessment of CI in the first place. In addition, we need to learn the influence of antipsychotic medication on CI. Especially promising might be the application of high intensity training, which seems to be feasible in patients with schizophrenia (63–66). Herbsleb et al. (67) showed in an case report that a 6-week high intensity training improved autonomic function substantially while no such effect was observed after 6 weeks of continuous training in a patient with schizophrenia. Most intriguingly, the previously observed pattern of autonomic dysfunction returned after 6 weeks of detraining. This study underlines the importance of the choice of the training method as well the introduction of long-lasting training concepts in psychiatry.

Some limitations need to be taken into account. This is an exploratory secondary analysis of the fitness testing results of previously published data (44–46). Though the initial study protocol (44) was not primarily designed to reveal CI it contained thorough fitness testing [executed by a trained sports scientist (KKV)] to objectively assess cardiovascular fitness levels in the included patients with schizophrenia before and after the exercise intervention. However, the obtained data allowed us to calculate the metabolic-chronotropic relationship and hence CI *post-hoc*. As outlined above, the presence of CI can detected regardless the protocol (continuous or step-wise increase of the workload) during formal exercise testing and a variety of exercise testing protocols can be used (37, 52). This is of high interest when re-analyzing fitness testing data of existing exercise studies to shed more light on the phenomenon of exercise intolerance in patients with schizophrenia and to determine to which extend CI might be a contributing factor. The sample size is relatively small, especially those of SZ-CI that underwent 12 weeks of continuous aerobic exercise training.

## Conclusion

Our results confirm the occurrence of CI in patients with multi-episode schizophrenia. Thorough fitness testing is necessary to reveal CI, but it does not seem to make a difference if the workload during the test is raised continuously or stepwise every 3 min. This should be taken into account when planning an exercise or lifestyle intervention study in patients with schizophrenia. Further studies are also needed to confirm CI in a prospective sample of patients with schizophrenia and to examine the underlying pathology of CI in patients with schizophrenia, i.e., by performing additional structural and functional examinations of the heart, as CI might rely to a specific cardiovascular pathophysiology in patients with schizophrenia. Furthermore, schizophrenia patients with CI do not seem to benefit as well as schizophrenia patients without CI from moderate continuous aerobic exercise training interventions, which may explain mixed results in this population in the literature. Larger, prospective randomized controlled clinical trials with different training interventions are urgently needed to address the topic of schizophrenia patients not responding to exercise and the relationship to the illness itself.

## Author Contributions

MH analyzed the data and wrote the first draft of the study. KK-V designed and performed the study and critically analyzed the data. TW, AH, and AS designed the study, gave critical comments, and revised the first draft of the study. PF and BM designed and performed the study, critically analyzed the data, gave critical comments, and revised the first draft of the study. HHWG gave critical comments and critically analyzed the data. K-JB designed the study, gave critical comments, critically analyzed the data, and revised the first draft of the study.

### Conflict of Interest Statement

The authors declare that the research was conducted in the absence of any commercial or financial relationships that could be construed as a potential conflict of interest.
